# Gene transcriptional expression of cerebral blood flow alterations in Parkinson's disease: a transcription-neuroimaging association study

**DOI:** 10.1016/j.nicl.2026.104033

**Published:** 2026-07-06

**Authors:** Jiaqi Cui, Qiane Yu, Haifeng Ran, Kexin Huang, Jie Hu, Tijiang Zhang

**Affiliations:** a Department of Radiology, The Affiliated Hospital of Zunyi Medical University, Medical Imaging Center of Guizhou Province, Zunyi 563000, China; b Department of Radiology, Kweichow Moutai Hospital, Renhuai 564500, China; c Department of Radiology, Union Hospital, Tongji Medical College, Huazhong University of Science and Technology, Wuhan 430022, China; d Bijie Medical College, Bijie 551700, China

**Keywords:** Parkinson's disease, Cerebral blood flow, Cognitive function, Gene expression, Enrichment analysis

## Abstract

**Aim:**

Despite the intimate link between cerebral blood flow (CBF) alterations and Parkinson's disease (PD) pathogenesis and cognitive decline, the precise pathophysiological mechanisms driving this relationship remain elusive. This study seeks to correlate changes in CBF with regional gene expression to advance mechanistic understanding of the disease.

**Methods:**

CBF group differences were first determined from ASL data in 53 PD and 40 healthy controls (HC) and subsequently correlated with cognitive scores (Dataset 1). A coordinate-based meta-analysis of published literature provided a second set of CBF differences (Dataset 2). Transcriptomic data from the Allen Human Brain Atlas were then correlated with hemodynamic patterns derived from each dataset to identify genes associated with CBF alterations across two independent datasets. The statistically significant genes from each cohort were intersected to obtain a common gene set, which subsequently underwent functional annotation and cell-type enrichment analysis. Finally, cross-modal spatial correlation was employed to investigate CBF changes associated with neurotransmitters.

**Results:**

In dataset 1, PD showed decreased CBF in frontal and occipital regions, but increased CBF in limbic and parietal areas. CBF variations in the hippocampus, parahippocampal gyrus, and postcentral gyrus correlated with cognitive scores (MMSE/MoCA). Dataset 2 revealed frontal region and angular gyri hypoperfusion. Integration with transcriptomic data identified an overlapping gene set, enriched in synaptic structure, plasticity, and energy metabolism pathways. Cellular enrichment showed predominant expression in excitatory/inhibitory neurons, microglia, and oligodendrocyte precursor cells. Neurotransmitter association analysis linked CBF alterations to the norepinephrine transporter (NET).

**Conclusion:**

By integrating transcriptomic and neuroimaging findings, this study suggests that CBF alterations may be linked to specific genes and biological processes in PD.

## Highlights

• Cerebral blood flow changes in Parkinson's disease linked to cognitive decline
• Frontal and hippocampal blood flow alterations highlight key vulnerable regions
• Multi-dataset validation integrates neuroimaging with brain-wide gene expression
• Synaptic and metabolic genes implicated in Parkinson's pathophysiology

## 1 Introduction

The clinical and economic burden of Parkinson's disease (PD), a disorder characterized by dopaminergic neuron loss, is growing in China due to increased prevalence from population aging and is further intensified by the absence of effective therapies (Kalia and Lang, 2015; Qi et al., 2021; Zheng et al., 2023). New research further indicates that PD may not be a unified condition; its pathological basis may be more complex, possibly involving neurodegenerative changes and disorders of multiple neurotransmitter systems (Müller-Nedebock et al., 2023; Yang et al., 2023; Teleanu I et al., 2022). However, the pathophysiology connecting macroscopic brain function to molecular alterations remains incompletely elucidated, necessitating deeper neurobiological insights for targeted intervention development.

Magnetic Resonance Imaging (MRI), being radiation-free and offering exceptional soft-tissue contrast, is indispensable for investigating chronic, progressive neurodegenerative diseases. Arterial spin labeling (ASL) offers a non-invasive, quantitative approach for measuring cerebral blood flow (CBF) with advantages in resolution and cost-effectiveness over traditional modalities (Petcharunpaisan et al., 2010; Iutaka et al., 2023). This technique revealed that the spatial distribution of hypoperfused regions in PD patients overlaps with metabolic deficits observed on fluorodeoxyglucose PET (Jia et al., 2018; Kamagata et al., 2011; Syrimi et al., 2017; Hosokai et al., 2009; Teune et al., 2014). CBF research in PD spans motor symptoms, non-motor symptoms (e.g., cognitive dysfunction, hallucinations, and depression), and disease subtypes (Yoo et al., 2024; Wang et al., 2022; Matsui et al., 2006a; Matsui et al., 2006b; Pan et al., 2025). Collectively, these observations support the role of CBF alterations as a potential biomarker for diagnosis and progression monitoring. However, current studies are largely confined to the “imaging-symptom” level and may lack cross-scale mechanistic explanations.

Neuroimaging serves as a critical bridge connecting genes, imaging phenotypes, and clinical symptoms, effectively linking macroscopic observations to microscopic mechanisms. By treating neuroimaging metrics as endophenotypes, imaging genetics offers higher sensitivity than conventional genome-wide association studies (GWAS) and enables the non-invasive identification of genetic associations through computational algorithms, thereby facilitating the development of integrated diagnostic biomarkers (Hashimoto et al., 2015; The Parkinson progression marker initiative (PPMI), 2011). Collectively, these attributes underscore the considerable potential of integrating neuroimaging with genetic data in elucidating the complex pathological mechanisms of PD. Recent studies have established correlations between gene expression patterns and various neuroimaging metrics (Mroczek et al., 2021), including R2 recovery time and cortical atrophy (Yu et al., 2017). In PD research, cortical atrophy patterns have also been linked to mitochondrial and metabolic gene pathways (Vo et al., 2023). However, current PD neuroimaging-genetic studies still face limitations: first, a lack of integration with other imaging modalities, such as CBF. Second, frequent constraints due to limited sample sizes or single-center study designs. Meta-analysis addresses these constraints by aggregating data across multiple studies, improving statistical power and result reliability. Moreover, its standardized protocols effectively reduce potential subjective biases.

**Fig. 1:**
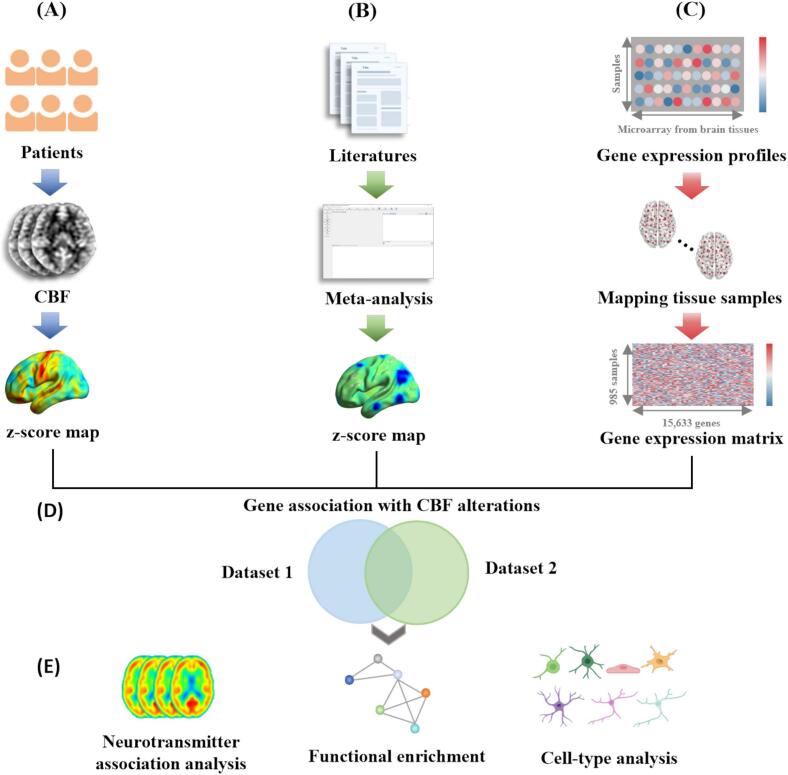
Study overview. (A) CBF images from our centre were pre-processed to yield group-difference z-statistic images. (B) After systematic retrieval and screening, published data were meta-analyzed with SDM-PSI to generate case-control z-statistic maps. (C) Transcriptomic profiling. The regional gene expression value was extracted from the allen human brain map to construct an expression matrix. (D) PLS regression linked CBF alterations to gene-expression data; significant genes in PLS1 from both datasets were identified, ranked, and intersected. (E) The overlapping gene set was subjected to functional and cell-type enrichment analyses. Concurrently, case-control CBF-difference maps from both datasets were spatially correlated with neurotransmitter atlases to identify transmitters linked to CBF alterations. PLS, Partial Least Squares; CBF, Cerebral Blood Flow.

Therefore, this study integrated two CBF datasets, one from our research center and another from a meta-analysis to determine the CBF alterations of PD patients through comparative analysis. We subsequently incorporated whole-brain transcriptomic data to investigate potential associations between CBF changes and gene expression patterns. Finally, functional enrichment and neurotransmitter correlation analyses were performed on the identified genes to elucidate the underlying pathological mechanisms of PD (Fig. 1 is a schematic diagram of this study).

## 2 Methods

### 2.1 Dataset 1

A cohort was recruited from the Department of Neurology at Affiliated Hospital of Zunyi Medical University between July 2023 and November 2024. PD diagnoses were confirmed based on the International Parkinson's and Movement Disorder Society (MDS) clinical criteria (Postuma et al., 2015) by two board-certified neurologists. Patients abstained from antiparkinsonian medications for ≥12 h before MRI acquisition. Obtained the written informed consent of all participants and the ethical approval of the Institutional Review Committee. The flowchart for including and excluding patients is shown in Fig. S1. Inclusion criteria included: (1) complies with the MDS clinical diagnostic criteria; (2) age ≥ 40 years; (3) right-handed. Exclusion criteria included: (1) comorbid neurodegenerative disorders; (2) history of deep brain stimulation or surgical interventions; (3) atypical parkinsonism; (4) MRI contraindications (claustrophobia, non-removable metal implants) or artifacts from severe head tremor/motion; (5) sensory impairments (deafness/blindness) or consciousness disorders. Motor symptom severity was quantified using the Movement Disorder Society-Unified Parkinson's Disease Rating Scale Part III (MDS-UPDRS-III). Global cognitive function was assessed via the Mini-Mental State Examination (MMSE) and Montreal Cognitive Assessment (MoCA). Inclusion criteria for Healthy controls (HC): (1) age ≥ 40 years; (2) right-handed. Exclusion criteria: (1) history of significant head trauma or intracranial surgery; (2) any diagnosed neurological or psychiatric disorder; (3) Contraindications for MRI examination.

### 2.2 MRI protocol

MRI examinations were performed using a 3.0 Tesla Discovery MR750w scanner (GE Healthcare) equipped with a 32-channel phased-array head coil. Participants were positioned supine with foam padding to minimize head motion and earplugs to attenuate acoustic noise. Structural imaging involved a three-dimensional T1-weighted volumetric sequence with the following parameters: field of view (FOV) = 256 × 256 mm^2^; repetition time (TR) = 5000 ms; echo time (TE) = 3.232 ms; inversion time (TI) = 450 ms; flip angle (FA) = 8°; slice thickness = 1 mm; interslice gap = 0.5 mm; total slices = 328. SDASL data were obtained via a background-suppressed 3D pseudocontinuous ASL sequence: FOV = 240 × 240 mm^2^; TR = 4752 ms; TE = 10.2 ms; TI = 2025 ms; FA = 111°; slice thickness = 5.0 mm; Post-Labeling Delay = 2025 ms; no interslice gap. Participants were instructed to remain still, breathe steadily, and keep their eyes closed during the scanning process.

### 2.3 Data preprocessing and analysis

CBF maps were derived from ASL data and pre-processed in MATLAB using SPM12 (http://www.fil.ion.ucl.ac.uk/spm). All CBF images were first converted to MNI space, and then converted to z-score by subtracting the global average of CBF and dividing them by the whole brain standard deviation. Subsequently, these standardized images were smoothed with a Gaussian kernel of 6 mm full-width at half maximum (FWHM).

### 2.4 Statistical analysis

Demographic and clinical data were analyzed using the Social Sciences version 29.0 statistical package (SPSS, Chicago, IL, USA). The analysis of classified variables adopts the chi-square test. For continuous variables, the normal distribution data adopts the independent sample *t-*test, and the non-normal distribution data adopts the Mann-Whitney *U* test. Multiple comparisons were corrected using the Gaussian random field (GRF) method (voxel-wise *p* = 0.001; cluster-level *p* = 0.05). We then conducted partial correlation analysis to evaluate the association between PD-related CBF changes and cognitive performance, controlling for age and gender.

### 2.5 Meta-analysis (Dataset 2)

#### 2.5.1 Search strategy

This systematic review was registered with PROSPERO (CRD42024611244). Literature was retrieved using PubMed and Web of Science. In addition, the list of references in the relevant articles and reviews is manually reviewed to identify any qualified studies that may be missed. There were no restrictions on the year of publication. The search strategy employed the query (“Parkinson's Disease” OR “Parkinson's disease” OR “Parkinsons disease” OR “Parkinson's” OR “Parkinson” OR “Shaking Palsy” OR “Paralysis Agitans” OR “PD”) AND (“Cerebrovascular Circulation” OR “Cerebral Blood Perfusion” OR “Brain Blood Flow” OR “Cerebral Circulation” OR “Cerebral Perfusion” OR “Blood Circulation” OR “arterial spin labeling” OR “ASL” OR “asl mri” OR “Cerebral Blood Flow” OR “CBF”) and was restricted to English publications. The final search was conducted on November 24, 2024. All identified records are imported into EndNote for reference management, in which duplicate records are deleted according to the title, author, and year of publication. After excluding the conference summary and duplicate entries, the rest of the articles were manually screened for the title and summary, and then the full text was evaluated according to the inclusion criteria. To ensure methodological rigor, 20% of the screened articles were randomly selected for independent review by another investigator. Any discrepancies between reviewers have been resolved by consensus. The literature selection process is summarized in Fig. S2.

### 2.6 Study selection and data processing

Inclusion: (1) Observational or longitudinal studies; (2) The study presents peak coordinates in stereotactic 3D space (Talairach or MNI); (3) *t*-value, z-score, or *p*-value are extractable from the reported data. Exclusion: (1) Animal studies, review articles, case reports/series, editorials, and conference abstracts; (2) Research on Parkinson's syndrome, drug- or toxin-induced Parkinsonism, other movement disorders (e.g. Huntington's disease), or studies involving other populations without Parkinson's disease; (3) Studies not related to cerebral blood flow; (4) Studies employing region of interest (ROI) analysis.

A meta-analysis of CBF differences between PD and HC was performed using SDM-PSI (v6.23, https://www.sdmproject.com), following established protocols (Radua et al., 2012). Peak coordinates from relevant studies were extracted and converted to t-values using the SDM platform. The analysis incorporated heterogeneity assessment using I^2^statistics (Higgins et al., 2003) and evaluated publication bias with Egger's test (Egger et al., 1997). Spatial optimization was applied using a Gaussian kernel (FWHM = 20 mm) and cluster thresholds (*p* < 0.005, clusters ≥10 voxels), corrected with threshold-free cluster enhancement and family-wise error rate (TFCE-FWER) (*p* < 0.05).

### 2.7 Associations with gene expression

#### 2.7.1 Gene expression data preprocessing

Correlation of gene expression and brain imaging leveraged gene expression data from the Allen Human Brain Atlas (AHBA), a high-resolution microarray resource spanning six neurotypical adult donors (Hawrylycz et al., 2012) (https://human.brain-map.org/static/download). Processing followed the validated abagen toolbox (Markello et al., 2021) workflow (https://www.github.com/netneurolab/abagen), implementing seven standardized procedures to generate a sample × gene expression matrix: (1) Probe reannotation: Microarray probes were re-annotated to genes using the updated mapping (Arnatkeviciute et al., 2019), discarding probes lacking reliable gene matches; (2) Intensity-based filtering: Probes with expression levels below background signal in >50% of samples were removed; (3) Probe selection for multi-probe genes: For genes targeted by multiple probes, probe selection was performed using the differential stability (DS) method (DS > 0.1). This approach computes the cross-region Spearman correlation of each probe's expression across donors and retains the probe with the highest average correlation, ensuring the selected probe exhibits the most consistent inter-donor expression pattern and minimizing the impact of inter-individual sampling variability; (4) Sample selection: Following previous sample-level studies (Cai et al., 2024), we retained only cortical and subcortical gray matter samples, ensuring spatial consistency with our gray matter constrained CBF maps; (5) Sample-level normalization (across genes): Expression values for each tissue sample were normalized across genes using the scaled robust sigmoid (SRS) method, which uses median and interquartile range to resist outlier effects and reduce donor specific batch artifacts; (6) Gene level normalization (across samples): After sample level normalization, expression values for each gene were normalized across samples using the same SRS method, ensuring comparability within and across donors; (7) Quality control and final sample selection: After filtering for probes, selecting gray matter samples, and excluding those with missing annotations or expression profiles more than two standard deviations from the mean interareal similarity, we retained 985 samples with complete transcriptomic coverage. The imaging analysis was performed on the retained 985 AHBA tissue samples and their corresponding spatial locations. The resulting gene expression matrix had dimensions of 985 samples × 15,633 genes, where each row corresponds to an individual tissue sample with known MNI coordinates and each column to a gene. This matrix was used for all subsequent analyses.

#### 2.7.2 Spatial correlation with gene expression profiles

First, the group-difference CBF maps derived from two independent datasets were normalized using z-transformation: dataset 1 was the in-house independent CBF dataset, and dataset 2 was the meta-analysis cohort. Subsequently, for each tissue sample, we extracted the mean z-score from the z-maps of both datasets within a sphere of 6 mm radius centered on the sample's MNI coordinate. Finally, partial least squares (PLS) correlation analysis was performed to examine the multivariate relationship between the CBF z-score vector and the sample-wise gene expression matrix across all tissue samples. Specifically, the CBF z-score vector was treated as the dependent variable Y, and the gene expression matrix (genes × samples) was treated as the independent variable X. The PLS analysis was used to identify linear combinations of gene expression components that maximally covary with CBF alterations, thereby revealing the gene expression components most strongly associated with the CBF changes.

We use BrainSMASH (Burt et al., 2020) to generate surrogate brain maps that preserve the spatial autocorrelation (SA) of a target map through a generative null modeling framework. First, the empirical map is randomly permuted, disrupting its spatial organization and any intrinsic intermodal relationships. Spatial autocorrelation is then reintroduced by smoothing the permuted map using a distance-dependent kernel (exponential decay kernel by default). To match the SA of the target map, the variogram of the smoothed map is computed and linearly regressed onto the target variogram. The smoothing strength, parameterized as the proportion of nearest neighbors, is iteratively optimized to minimize the sum of squared errors in the variogram fit. The optimal smoothing parameter is used to generate each surrogate map, and this entire procedure is repeated independently for every null map in the ensemble. The resulting surrogate maps exhibit empirical variograms that closely approximate those of the original brain map, enabling statistically valid inference under preserved spatial autocorrelation. Each gene underwent 10,000 resampling iterations to generate PLS weights, and z-score were calculated to assess contribution. Only genes with significant z-score (*p* < 0.05, FDR-corrected) were retained for further analysis. Finally, overlapping genes from the two models were selected for enrichment analysis. All preprocessing and gene association steps for the unsmoothed dataset 1 (the validation dataset) were performed identically to those described above.

#### 2.7.3 Gene enrichment and cell-type analysis

Gene enrichment was performed using Metascape (Zhou et al., 2019) (https://metascape.org/gp/index.html) for functional enrichment of genes with PLS weights (*p* < 0.05, FDR-corrected). Gene Ontology (GO) and KEGG databases were employed, and enrichment analysis was conducted against the background of the entire genome. The *p*-value was calculated by the cumulative hypergeometric distribution, and the multiple comparison was corrected by the Benjamini-Hochberg method (*p* < 0.01). The top 20 enriched pathways for each directional association were reported for further investigation.

Through integration of five independent single-cell RNA sequencing datasets from human post-mortem brain tissue, we established gene sets representing distinct cell types. This comprehensive approach incorporated 58 initial cellular annotations to minimize technical biases. To resolve nomenclature and compositional discrepancies, cell types were harmonized into seven consensus classes: microglia, endothelial cells, oligodendrocyte precursor cells, oligodendrocytes, astrocytes, excitatory neurons, and inhibitory neurons (Seidlitz et al., 2020). We linked CBF-associated genes to these unified cellular categories by assessing the overlap between cell-type gene sets and PLS1-weighted gene sets, with the significance of the overlap evaluated using permutation testing. The number of overlapping genes in each cell type was determined (Romero-Garcia et al., 2020) (*p* < 0.05, FDR-corrected). Next, functional enrichment analysis is carried out to investigate the biological pathways related to the genes of each cell type (*p* < 0.05, FDR-corrected).

### 2.8 JuSpace-based spatial correlation mapping of neurotransmitter receptors

Cross-modal spatial correlation analysis was conducted using the JuSpace toolbox (Dukart et al., 2021), following the detailed workflow: maps of CBF z-score for differences between groups, extracted from both dataset 1 and dataset 2, were correlated spatially with neurotransmitter receptor templates derived from PET/SPECT. Each PET map and its source are detailed in the file “Sources_templates_release.txt” distributed with JuSpace and in the original toolbox publication (Dukart et al., 2021). These include the dopamine transporter map derived from ^123^I-Ioflupane SPECT of 30 HC available through NITRC, the norepinephrine transporter map from [^11^C]MRB PET of 10 HC, the serotonin transporter map from [^11^C]DASB PET of 210 HC from the Cimbi database, and the μ-opioid receptor map from [^11^C]Carfentanil PET of 89 HC obtained from Neurovault. To assess the spatial association between CBF alterations and neurotransmitter systems, we performed voxel-wise Pearson correlation analyses between the group-level CBF maps and each PET-derived receptor template. All neurotransmitter maps distributed with JuSpace are publicly available group-level templates. We used a spatial permutation approach implemented in JuSpace to assess statistical significance. For each analysis, we generated 10,000 surrogate maps that matched the spatial autocorrelation of the original PET templates by permuting the data and reintroducing spatial smoothness. The correlation between the CBF maps and each surrogate map was computed to create an empirical null distribution, and the observed correlation was then evaluated against this distribution to derive *p*-values. (*p* < 0.05, FDR-corrected).

## 3 Results

### 3.1 Demographic and clinical characteristics

#### 3.1.1 Dataset 1

The study included 93 participants: 53 PD patients (24 male, 29 female; age 61.00 ± 8.91 years) and 40 HC (18 male, 22 female; age 59.13 ± 6.78 years). No significant intergroup differences were observed in age (*t* = 1.109, *p* = 0.270), sex distribution (*χ*^*2*^ = 0.001, *p* = 0.978), or education (*t* = 0.373, *p* = 0.895). Detailed demographic and clinical metrics are summarized in Table 1.

**Table 1:** Demographic and clinical characteristics of PD and HC groups in dataset 1.

Characteristics	PD	HC	*t/*χ2	*p-*value
Number of subjects	53	40	–	–
Gender (female/male)	29/24	22/18	0.001	0.978^a^
Age (year)	61.000 ± 8.914	59.125 ± 6.779	1.109	0.270^b^
Education (year)	7.462 ± 3.848	8.125 ± 3.065	−0.895	0.373^b^
Duration(year)	5.226 ± 4.678	–	–	–
MMSE(score)	23.415 ± 5.617	27.875 ± 2.409	−4.701	0.000^b^
MoCA(score)	16.962 ± 6.522	23.150 ± 4.538	−5.133	0.000^b^
UPDRS III(score)	22.830 ± 21.825	–	–	–

PD, Parkinson's Disease; HC, Healthy control; MMSE, Mini-mental State Examination; MoCA, Montreal Cognitive Assessment; UPDRS, Unified Parkinson's Disease Rating Scale.

a Chi-square test.

b Two-sample *t*-test.

#### 3.1.2 Meta-analysis (Dataset 2)

Following a preliminary review of titles and abstracts, 22 articles were deemed eligible. After reading the full text, 13 studies were included. In 13 CBF studies, the CBF differences between 413 PD patients (255 males, 158 females, with an average age of 63.993 years) and 392 HC (200 males, 192 females, with an average age of 63.293 years) were reported. Detailed information are shown in Table S1.

### 3.2 Cerebral blood flow changes

**Fig. 2:**
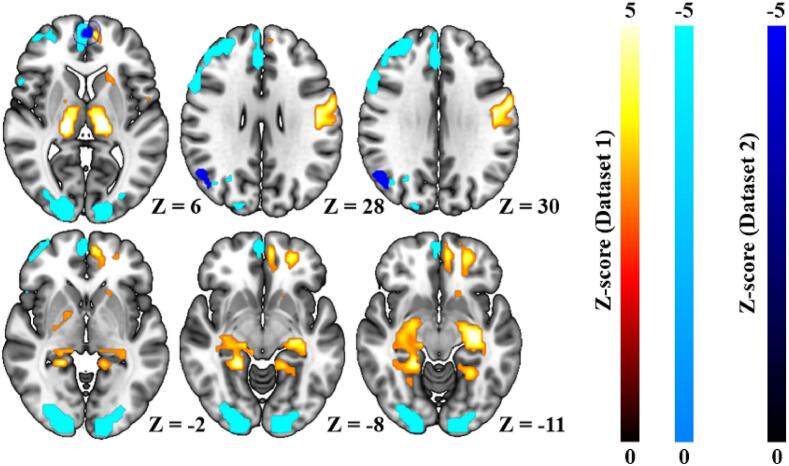
CBF differences between the PD and HC cohorts. Light-blue and orange denote regions with pronounced CBF changes in dataset 1 (light-blue: decreased CBF; orange: increased CBF); dark-blue indicates areas of reduced CBF in dataset 2. The region outlined in blue delineates the brain area where the two datasets exhibit convergent alterations. PD, Parkinson's Disease; HC, Healthy Control; CBF, Cerebral Blood Flow.

In dataset 1, PD showed decreased CBF in the bilateral superior frontal gyri, right inferior frontal gyrus, right middle frontal gyrus, right medial frontal gyrus, right lingual gyrus, right inferior occipital gyrus, right anterior cingulate gyrus, and right calcarine sulcus cortex. Increased CBF was observed in the left hippocampus, left parahippocampal gyrus, left middle frontal gyrus, left superior frontal gyrus, left medial frontal gyrus, left primary sensorimotor cortex, and left postcentral gyrus. In dataset 2, decreased CBF was found in the bilateral superior frontal gyri and right angular gyrus, with no significant hyperperfusion detected. The brain regions with significant between-group CBF differences are summarized in Table 2 and Fig. 2. Spatial correlation analysis showed a correlation between the two datasets in their z-score maps of CBF differences (*r* = 0.301, *p* < 0.001) (Fig. S4A). In dataset 1, the average correlation within the null distribution was 0.245; in dataset 2, the average correlation was 0.239.

**Table 2:** Brain areas exhibiting altered CBF in PD versus HC in two datasets.

Brain areas (peak coordinates)	MNI	z-score	*p*-value	Voxel	Cluster
PD < HC in CBF (Dataset 1)					
Right inferior frontal gyrus	54, 17, 28	−4.128	<0.001	462	Right middle frontal gyrus
					Right inferior frontal gyrus
Right medial frontal gyrus	3, 59, −8	−4.909	<0.001	297	Right superior frontal gyrus
					Right anterior cingulate gyrus
					Left superior frontal gyrus
Right lingual gyrus	24, −85, −2	−3.801	<0.001	281	Right lingual gyrus
					Right inferior occipital gyrus
					Right calcarine sulcus cortex
PD > HC in CBF (Dataset 1)					
Left paracentral lobule	−18, −22, 64	4.636	<0.001	381	Left paracentral lobule
					Left precentral gyrus
Left hippocampus	−30, −19, −11	4.63	<0.001	351	Left hippocampus
					Left inferior temporal gyrus
					Left parahippocampal gyrus
Left middle frontal gyrus	−24, 50, −11	4.324	<0.001	349	Left superior frontal gyrus
					Left middle frontal gyrus
					Left medial frontal gyrus
Left postcentral gyrus	−48, −10, 28	4.469	<0.001	312	Left postcentral gyrus
					Left precentral gyrus
PD < HC in CBF (Dataset 2)					
Right angular gyrus	50, −64, 32	−6.156	<0.001	152	Right angular gyrus
					Right middle occipital gyrus
Left superior frontal gyrus	0, 58, 6	−5.753	<0.005	66	Left superior frontal gyrus
					Right superior frontal gyrus

PD, Parkinson's Disease; HC, Healthy Control; CBF, Cerebral Blood Flow, MNI, Montreal Neurological Institute.

#### 3.2.1 Correlation analysis of CBF with cognitive scale scores

**Fig. 3:**
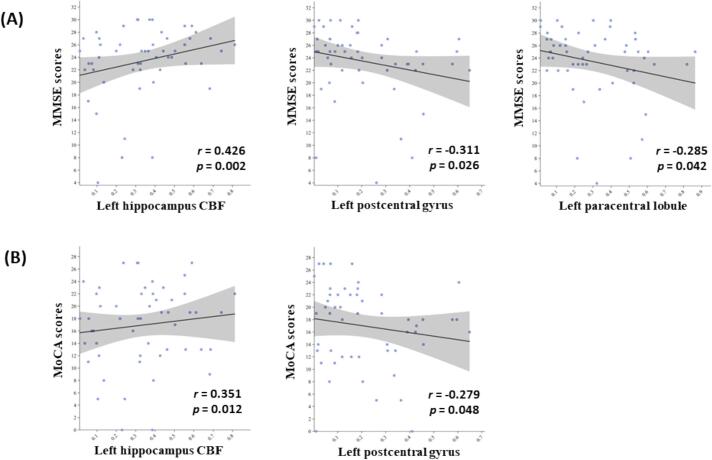
The correlation between regional CBF values and cognitive performance (MMSE/ MoCA). (A) CBF in the left hippocampus correlates positively with MMSE scores (*r* = 0.426, *p* = 0.002), whereas CBF in the left precentral gyrus (*r* = −0.285, *p* = 0.042) and postcentral gyrus (*r* = −0.311, *p* = 0.026) correlates negatively with MMSE scores. (B) CBF in the left hippocampus correlates positively with MoCA scores (*r* = 0.351, *p* = 0.012); CBF in the left precentral gyrus correlates negatively with MoCA scores (*r* = −0.279, *p* = 0.048). CBF, Cerebral Blood Flow; MMSE, Mini-Mental State Examination; MoCA, Montreal Cognitive Assessment.

The CBF values of the left hippocampus (*r* = 0.426, *p* = 0.002), left postcentral gyrus (*r* = −0.311, *p* = 0.026), and left paracentral lobule (*r* = −0.285, *p* = 0.042) are significantly correlated with the MMSE cognitive scale scores, as shown in Fig. 3A. The CBF values of the left hippocampus (*r* = 0.351, *p* = 0.012) and left postcentral gyrus (*r* = −0.279, *p* = 0.048) are significantly correlated with the MoCA cognitive scale scores, as shown in Fig. 3B.

### 3.3 Association with gene expression

**Fig. 4:**
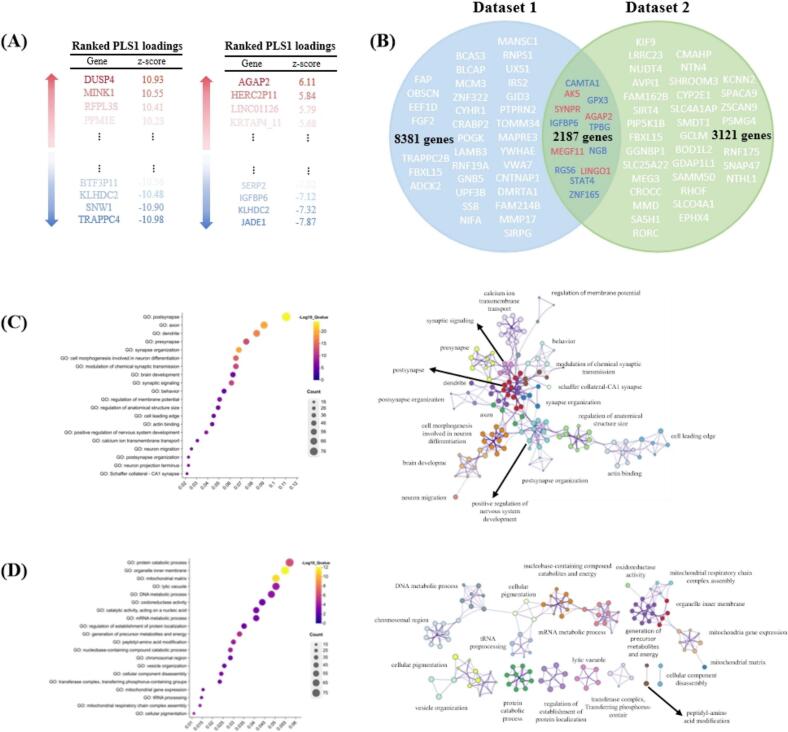
Gene expression profiles associated to CBF alterations. (A) Ranked PLS1 loadings (left panel: dataset 1; right panel: dataset 2). (B) Top-ranked and overlapped genes from two datasets. Functional enrichment analysis of top-ranked and overlapped genes (from B) with PLS1 + (C) and PLS1− (D) weights (*p* < 0.05, FDR-corrected). Left Panel: The diameter of each circle denotes the quantity of genes associated with its corresponding term. Right Panel: Metascape enrichment network visualization shows the intra-cluster and inter-cluster similarity of enrichment items. Each term is represented by a circle. The size of the circle is directly proportional to the number of genes contained in each term, and the cluster is represented by colour (nodes of the same colour belong to the same cluster). PLS, Partial Least Squares; CBF, Cerebral Blood Flow.

By using the PLS regression method, we found that the first PLS component (PLS1) can explain 17.14% (*p*_dataset1_ = 0.045, corrected for spatial autocorrelation) and 9.77% (*p*_dataset2_ = 0.0006, corrected for spatial autocorrelation) of the spatial changes in CBF in the case-controlled difference diagram based on the weighted gene expression pattern (Fig. S3). A significant positive correlation was observed between the case-control CBF difference z-score maps of the two datasets (*r* = 0.415, *p* < 0.001) (Fig. S4 A). Additionally, both datasets showed significant positive correlations between their respective z-score maps and regional PLS1 scores (dataset 1: *r* = 0.415, *p* < 0.001; dataset 2: *r* = 0.313, *p* < 0.001) (Fig. S4B). The average variance explained (R (Qi et al., 2021)) under the null was 0.062 and 0.059 for dataset1 and dataset2, respectively. Subsequently, all genes were ranked based on their z-scores, which led to the identification of 8381 genes in dataset 1 that showed significant contributions to PLS1 (*p* < 0.05, FDR-corrected). The PLS1+ gene set contained 3489 genes, while the PLS1− set consisted of 4892 genes. In dataset 2, 3121 genes showed significant contributions (*p* < 0.05, FDR-corrected), of which the PLS1+ set included 1284 genes and the PLS1− set comprised 1837 genes (Fig. 4A). Finally, by comparing the significant genes across both datasets, we obtained an overlapping gene set of 2187 genes (Fig. 4B). This common set contained 789 genes in the PLS1+ set and 1398 genes in the PLS1− set, and several top-ranking genes within this intersecting set (e.g., *AK5*, *PTPRN2*, *GRIN2B* etc.) were found to be significantly correlated with CBF alterations in PD.

Subsequently, we validated this approach by associating the unsmoothed dataset 1 (validation dataset) with gene expression data. Using PLS regression, we found that the PLS1 explained 14.73% of the spatial variance in CBF based on the weighted gene expression pattern from the case–control difference map (after correction for spatial autocorrelation, *p* = 0.003; Fig. S5). The validation analysis further revealed a total of 7839 significant contributing genes (*p* < 0.05, FDR-corrected), of which 2063 overlapped with those from dataset 2. Overlap with the final gene set from the main results (2187 genes) included 2032 genes, corresponding to an overlap rate of 92.91% (Fig. S6).

**Fig. 5:**
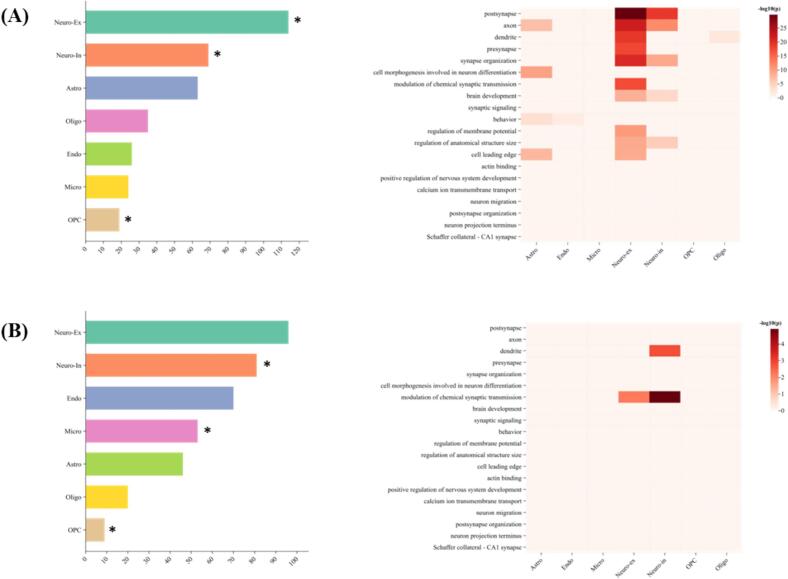
The cell type specificity of the genes associated to CBF alterations. Cell-type enrichment analysis of overlapped genes in PLS1 + (A) and PLS1− (B) gene set. Left Panel: The number of overlapping genes in each cell type. Right Panel: Genetic ontology terms enriched for alterations in CBF-related genes in cell types. * Indicates a significant correlation (*p* < 0.05).

Using Metascape, we separately analyzed PLS1+ and PLS1− gene sets. The PLS1+ gene set was enriched in GO biological processes related to postsynapse, axon, dendrite, and presynapse, but showed no KEGG pathway enrichment (Fig. 4C). In contrast, the PLS- gene set was enriched in processes such as protein catabolic process, organelle inner membrane, mitochondrial matrix, and lytic vacuole, also no KEGG pathways (Fig. 4D). Cell enrichment analysis showed that PLS1+ genes were significantly enriched in excitatory neurons (count = 114, *p* = 0.009), inhibitory neurons (count = 69, *p* = 0.007), and oligodendrocyte precursor cells (OPCs) (count = 19, *p* = 0.000). Conversely, PLS1- genes showed significant enrichment in inhibitory neurons (count = 81, *p* = 0.000), microglia (count = 53, *p* = 0.040), and OPCs (count = 9, *p* = 0.000). Further analysis showed that the PLS1+ gene in neural cells was linked to 12 gene ontology terms (e.g., “postsynapse” and “synapse organization”), while the PLS1− genes were associated with only two terms (“modulation of chemical synaptic transmission” and “axon”) (Fig. 5).

### 3.4 Neurotransmitter receptors

**Fig. 6:**
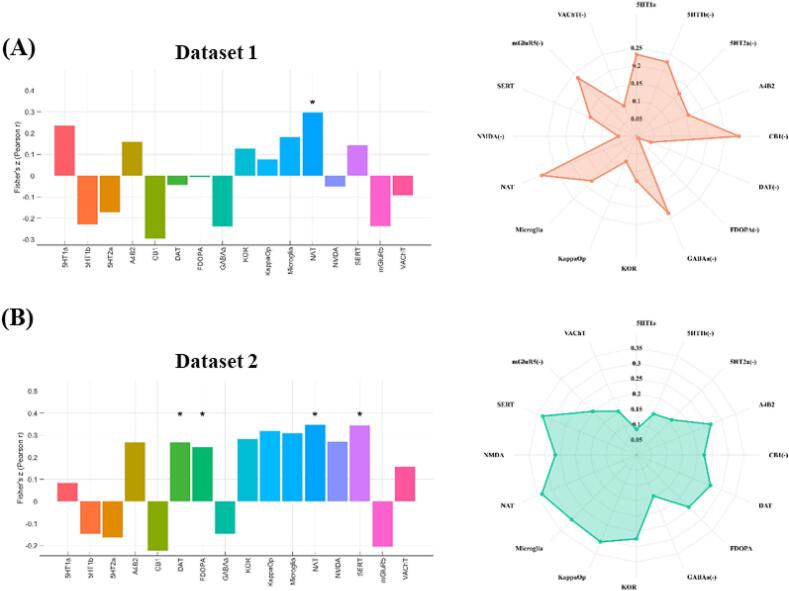
Analysis of neurotransmitters. (A) Left Panel: Neurotransmitters related to the CBF changes in dataset 1. Right Panel: R values of the results for the neurotransmitters are presented. (B) Left Panel: Neurotransmitters related to the CBF changes in dataset 2. Right Panel: R values of the results for the neurotransmitters are presented. The negatively correlated neurotransmitters are marked with (−). * Indicates a significant correlation (*p* < 0.05). NAT = NET, Noradrenaline Transporter. DAT, Dopamine Transporter; FDOPA, ^18^F-DOPA; SERT, Serotonin Transporter.

Neurotransmitter atlas correlation analysis further revealed heterogeneous neurotransmitter associations across datasets. In dataset 1, the noradrenergic atlas showed a significant spatial correlation with CBF differences between the PD and HC groups (norepinephrine transporter (NET), *r* = 0.288, *p* = 0.018). In dataset 2, multiple neurotransmitter systems were implicated: CBF differences were significantly associated with the dopaminergic atlas (dopamine transporter, *r* = 0.261, *p* = 0.026; ^18^F-DOPA, *r* = 0.241, *p* = 0.036), serotonergic atlas (serotonin transporter, *r* = 0.331, *p* = 0.002), and again with the NET (*r* = 0.334, *p* = 0.004) (Fig. 6A-B).

## 4 Discussion

This study investigated potential mechanisms of CBF alterations in PD by integrating two CBF datasets with transcriptomic data from the AHBA. Analysis of both CBF datasets showed that CBF changes in dataset 1 predominantly involved the frontal lobe, hippocampus, and primary motor cortex, whereas those in dataset 2 were largely localized to the superior frontal gyrus and angular gyrus. This analysis identified genes enriched in key biological processes, particularly those involving synaptic structure, plasticity, and energy metabolism. These genes are proposed as potential drivers of the observed CBF changes in PD. Additionally, neurotransmitter correlation analysis revealed that the NET is linked to the observed CBF differences. Thus, this work advances our understanding of PD pathological mechanism by dissecting CBF-associated mechanisms across multiple biological scales, provides a critical reference for future mechanistic and therapeutic research.

In this study, regions exhibiting decreased CBF were predominantly located in the frontal lobe, a finding that aligns with earlier studies (Wang et al., 2022; Huang et al., 2007). Superior frontal gyrus as an important component of the executive control network, serves critical functions in supporting working memory, executive control, and sustained attention. Consequently, the observed hypoperfusion in this region may signify an early dysregulation within this functional network. Notably, we consistently observed CBF reductions in the superior frontal gyrus across both independent datasets, thereby lending further support to the existing theory. In contrast to the hypoperfusion pattern, our data demonstrate a significant increase in CBF within the hippocampal region, which was positively correlated with scores on both the MMSE and MoCA. This hippocampal hyperactivation may reflect an underlying pathophysiological mechanism, a notion supported by previous studies (Dang-Vu et al., 2012). Evidence indicates that individuals with objectively-defined subtle cognitive decline show heightened hippocampal CBF compared to both cognitively unimpaired and mild cognitive impairment groups (Thomas et al., 2021), This specific perfusion pattern suggests that, when confronted with early pathological challenges, the brain may activate compensatory processes within the neurovascular unit, increasing local blood flow to sustain synaptic function and neural network integrity in response to early neurodegenerative metabolic demands. Complementary studies suggest involvement of neuroinflammatory pathways and vasodilatory signaling, further supporting this compensatory framework (Araújo et al., 2022; Zhang and Gao, 2022; Alsop et al., 2008). Thus, early CBF elevation represents a plausible adaptive mechanism to maintain cognitive function under pathological stress. The observed disparities in CBF patterns between datasets likely reflect the known heterogeneity of PD, attributable to variations in disease stage, clinical subtype, compensatory mechanisms, and other pathophysiological processes (Wang et al., 2025). Nevertheless, the consistent involvement of specific brain regions across both datasets strengthens the validity of our conclusions and provides supporting evidence for subsequent investigations into PD-related CBF alterations.

By combining CBF with gene expression data in the human brain, we find that PD may affect normal brain functions through multi-gene and multi-pathway mechanisms. Among the genes significantly related to our results, *AK5* serves as a key regulatory gene in PD pathology. As a member of the adenylate kinase family, it catalyzes the phosphoric acid transfer between AMP and ADP, which is a critical process for maintaining cell energy homeostatic and neuronal energy supply (Fujisawa, 2023). Purine metabolism dysregulation in PD disrupts ATP/ADP balance and compromises neuronal energetics (Garcia-Esparcia et al., 2015; Siddiqui et al., 2024). Therefore, the observed *AK5* upregulation may reflect a compensatory reaction to energy disruption and could also be associated with PD-related neuroinflammation. *PTPRN2* plays a central role in synaptic signaling regulation. Its coding protein is located in the synaptic vesicles and directly participates in the packaging and release of neurotransmitters, especially dopamine. Its dysfunction is the basis of the core motor disorder of PD. Animal studies have validated the functional importance of *PTPRN2*, and human investigations using blood and brain tissue have further linked this gene to PD motor symptom progression (Nishimura et al., 2009; Chuang et al., 2019; Grünblatt et al., 2004). Our gene enrichment analysis identified multiple synaptic-related GO pathways, consistent with *PTPRN2*'s regulatory role in synaptic function. This coherence not only reinforces the validity of our genomic findings but also supports the hypothesis that *PTPRN2* contributes to PD motor progression through synaptic dysregulation. *GRIN2B* dysfunction is related to the motor and cognitive impairments of PD. It encodes the NR2B subunit of the NMDA receptor, which is implicated in glutamate-mediated excitatory poisoning, which contributes to neuroinflammation and neuron loss, thus exacerbating PD symptoms (Schito et al., 1997). These mechanisms are further supported by animal studies (Canerina-Amaro et al., 2019; Yan et al., 2020; Ferreira et al., 2017). Although *GRIN2B* is polymorphic, its risk genotypes demonstrate higher prevalence in late-onset PD (onset age > 50 years) compared to controls, suggesting a specific role in late-onset disease susceptibility (Cui et al., 2024). This observation is consistent with our cohort, in which most patients also exhibited onset after age 50, thereby reinforcing this association.

In the ontological classification of cell-type specific features of upregulated gene sets, genes with higher weights are enriched in excitatory neurons, inhibitory neurons, and oligodendrocyte precursor cells (OPCs). In contrast, in the downregulated gene set, genes with higher weights are enriched in inhibitory neurons, microglia, and OPCs. The phenomenon of downregulated genes being enriched in microglia during the progression of PD may reflect the transition of PD from a “homeostatic” state to a “disease-associated” state. There is evidence suggesting that microglia may play a role in the clearance or spread of α-synuclein through mechanisms such as phagocytosis and exosome release. However, in PD and other neurodegenerative disorders, microglia exhibit a distinct phenotype, known as “disease-associated microglia” (Gao et al., 2023). Therefore, once microglia transition into a disease-associated state, this could potentially reduce their protein clearance ability, indirectly promoting the accumulation of pathological proteins and exacerbating neuroinflammation and dopaminergic neuron death. Additionally, upregulated genes are only enriched in excitatory neurons, whereas both upregulated and downregulated genes are enriched in inhibitory neurons. This suggests that the pathophysiology of PD may involve a complex imbalance between inhibitory and excitatory neural networks. Downregulation of inhibitory neurons in the prefrontal cortex likely exacerbates cognitive impairment, whereas their upregulation in the basal ganglia may attempt to dampen excessive excitation within motor circuits. Together, these processes disrupt neural circuit stability, providing a cellular basis for the co-occurrence of motor and cognitive deficits in PD and corresponding to the observed mechanism of prefrontal hypoperfusion. Furthermore, genes were not significantly enriched in mature oligodendrocytes (OLs), while both upregulated and downregulated genes were significantly enriched in OPCs. This finding raises the possibility that white matter abnormalities in PD may not stem from dysfunction in mature oligodendrocytes themselves (Yang et al., 2023; Han et al., 2022), but rather from impaired differentiation capabilities of oligodendrocyte precursor cells. The relative transcriptional silencing of oligodendrocytes could further mask functional decline, while OPCs appear to act as early responders in the pathological process.

Across both datasets, alterations in CBF were significantly associated with the NET. NET acts as the “off switch” for both central and peripheral norepinephrine synapses. Multiple research studies have shown a significant reduction in the density of the NET in PD. This decrease is strongly correlated with both motor and cognitive impairments (Laurencin et al., 2024). Additionally, NET plays a crucial role in enhancing the toxicity of α-synuclein by modulating the clearance rate of norepinephrine (Paredes-Rodriguez et al., 2020). As such, it may serve as a key regulatory factor in the progression of PD. Moreover, a NET-PET study comparing distinct freezing phenotypes in PD revealed that patients exhibiting levodopa-responsive freezing of gait (FOG) exhibited a marked reduction in thalamocortical NET binding (Mckay et al., 2023). The magnitude of this deficit scaled directly with freezing severity, suggesting that norepinephrine terminal degeneration might as a core mechanism and a putative therapeutic target for this FOG subtype, also that its associated metabolic pathways may also contribute to PD subtype classification or disease progression. These findings provide support for investigating the interactions between CBF changes and neurotransmitter activity in PD, offering new perspectives for exploring the potential mechanisms underlying the disease.

There are some limitations in this study: First, although dual-cohort comparisons enhance robustness, subtype stratification and longitudinal follow-up were absent. CBF is inherently dynamic, and the differences between different individuals (due to different disease processes or subtypes) may lead to an increase in variation values; therefore, planned longitudinal tracking or subtype-based division can alleviate this limitation. Secondly, despite a 12-h medication-free interval, prior dopaminergic exposure cannot be fully negated as a confounder; enrolling drug-naïve, newly diagnosed patients would offer a cleaner assessment. While the sample-level approach preserves the genuine anatomical origin of each tissue specimen, the AHBA dataset is markedly left-hemisphere dominant: only two of the six donors contributed right-hemisphere samples. Consequently, our results are predominantly weighted toward left-hemisphere transcriptional patterns, limiting their generalizability to the right hemisphere and warranting careful interpretation. Additionally, as CBF maps derive from in-vivo imaging while transcriptomic profiles originate from post-mortem tissue, interpretations hinge solely on spatial correspondence and should therefore be treated with caution. Furthermore, as this study focuses on potential associations between PD-related CBF alterations and transcriptomic profiles of the healthy brain, the findings should be interpreted with caution. The findings related to neurotransmitters also warrant further validation using more rigorous methods to confirm their stability and generalizability. Finally, because the CBF maps were Gaussian-smoothed, the observed spatial associations may partly reflect shared spatial autocorrelation. Future studies should consider using unsmoothed data to better assess biological specificity.

## 5 Conclusion

In this study, alterations in CBF in PD were primarily observed in the frontal lobe and hippocampus. These changes are thought to likely reflect impairments in patients' cognitive functions, along with potential compensatory mechanisms. By employing imaging transcriptomics and neurotransmitter-related analyses, we further identified that such CBF variations may be associated with pathways involved in synaptic structure and function, cellular energy, and substance metabolism. In addition, significant correlations were observed with excitatory and inhibitory neurons, microglia, oligodendrocyte precursor cells, and the norepinephrine transporter. Overall, our study provides new insights into the molecular and biological processes that may underlie the pathophysiological mechanisms of PD.

## Data sharing

Data of this research are available from the corresponding author upon reasonable request.

## Ethics approval and permissions

This study was approved by the Ethics Committee of the Affiliated Hospital of Zunyi Medical University (Ethics approval number: KLL-2023-015). Permission to use the Montreal Cognitive Assessment (MoCA) was obtained from the official MoCA organization (https://mocacognition.com/).

## CRediT authorship contribution statement

**Jiaqi Cui:** Writing – review & editing, Writing – original draft, Visualization, Validation, Software, Resources, Methodology, Investigation, Formal analysis, Data curation, Conceptualization. **Qiane Yu:** Writing – review & editing, Writing – original draft, Visualization, Supervision, Software, Methodology, Formal analysis, Data curation, Conceptualization. **Haifeng Ran:** Visualization, Validation, Supervision, Software, Methodology. **Kexin Huang:** Visualization, Supervision. **Jie Hu:** Writing – review & editing, Writing – original draft, Visualization, Validation, Supervision, Software, Methodology, Investigation, Formal analysis, Conceptualization. **Tijiang Zhang:** Writing – review & editing, Writing – original draft, Supervision, Project administration, Methodology, Funding acquisition, Conceptualization.

## Funding

This work was supported by the 10.13039/501100012166National Key Research and Development Program of China (No.2022YFC2009904), Construction of Scientific and Technological Innovation Talent Team of Functional Imaging and Artificial Intelligence Application Research in Guizhou Province (grant No. QianKeHeRenCai CXTD [2025] 047), and Intelligent Medical Imaging Engineering Research Center of Guizhou Higher Education Institutions project (Grant No. Qianjiaoji [2023] 038), and the Zunyi Science and Technology Innovation Talent Team (Grant No. ZSKRC [2024]) 5).

## Declaration of competing interest

The authors declare that they have no known competing financial interests or personal relationships that could have appeared to influence the work reported in this paper.

## Data Availability

Data will be made available on request.
