# Supplementary material for: Gene transcriptional expression of cerebral blood flow alterations in Parkinson's disease: a transcription-neuroimaging association study
Source: Neuroimage Clin. 2026 Jul 6;51:104033. doi: 10.1016/j.nicl.2026.104033 (PMC13382092; doi:10.1016/j.nicl.2026.104033)
Supplement: Supplementary file 1 — Supplementary material [file mmc1.docx]

**Supplementary Information for**

**Cerebral Blood Flow Changes Assessed by ASL MRI and Their Transcriptional Correlates in Parkinson’s Disease**

Jiaqi Cui^a,1^, Qiane Yu^a,b,1^, Haifeng Ran^a^, Kexin Huang^a^, Jie Hu^c,*^, Tijiang Zhang^a^_’_^d,^*

^a^ Department of Radiology, the Affiliated Hospital of Zunyi Medical University, Medical Imaging Center of Guizhou Province, Zunyi 563000, China.

^b^ Department of Radiology, Kweichow Moutai Hospital, Renhuai 564500, China

^c^ Department of Radiology, Union Hospital, Tongji Medical College, Huazhong University of Science and Technology, Wuhan 430022, China.

^d^ Bijie Medical College, Bijie, 551700, China.

^1^ These authors contributed equally to this work as co-first authors.

^*^**Corresponding author**

Tijiang Zhang

Email: [tijzhang@163.com](mailto:tijzhang@163.com)

<Tel:+86-13595246527>

Hu Jie

Email: [hujie917@hotmail.com](mailto:hujie917@hotmail.com)

[Tel:+86-](Tel:+86-13595246527)17785328741

**Supplementary Results**

**Figure S1. Flowchart of the patient’s inclusion process**


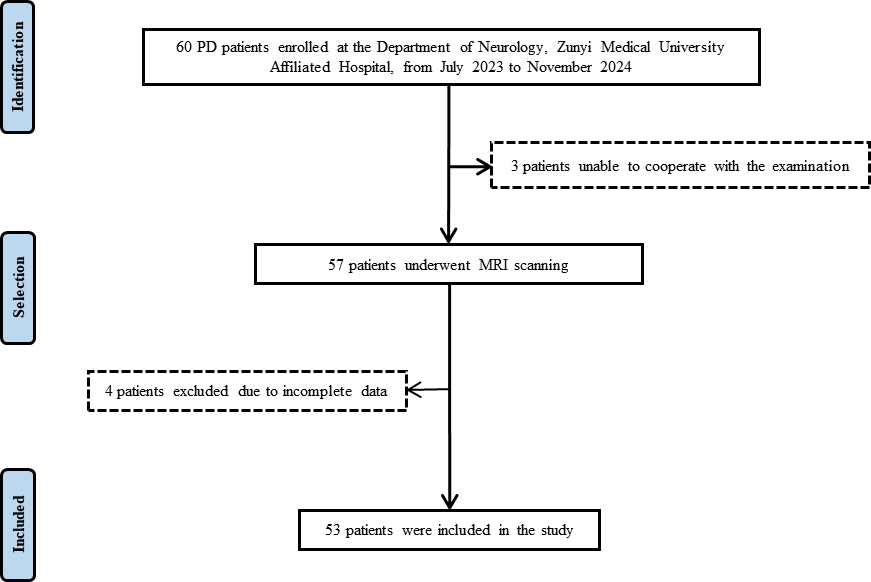


**Figure S2. Flowchart of the literature inclusion process**


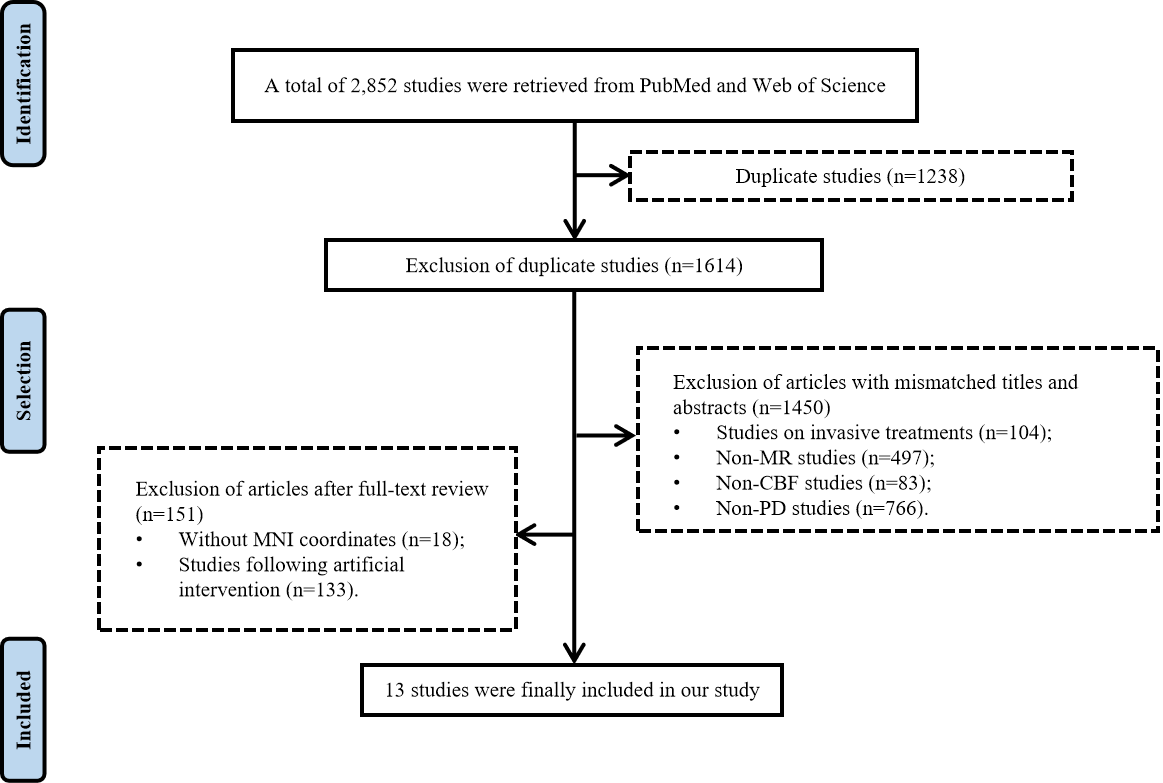


**Figure S3. Explained ratios for the first 10 components from the partial least squares (PLS) regression analysis**


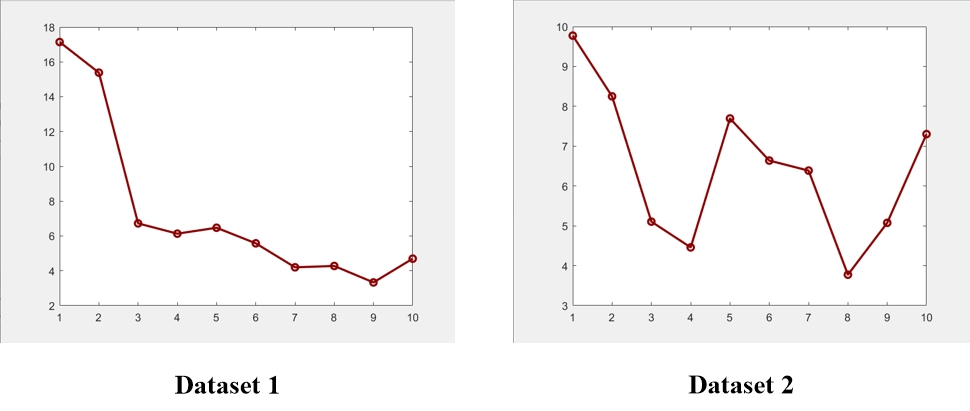


**Figure S4. Correlation analysis**


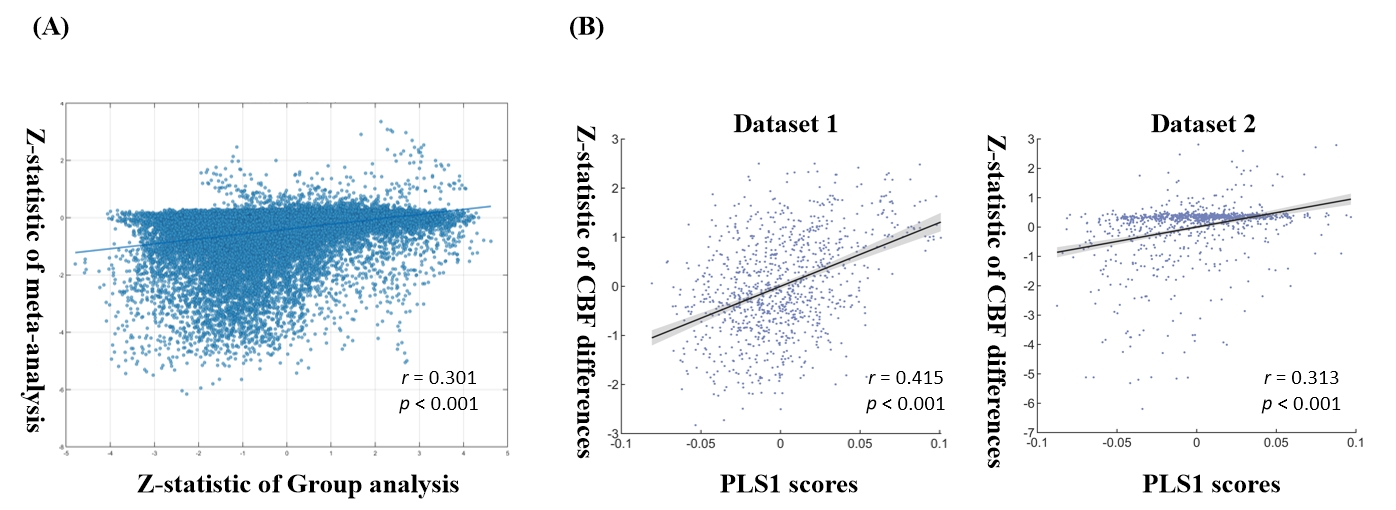


1. Scatter plot illustrating the correlation between z-statistics across the two datasets (*r* = 0.301, *p* < 0.001). (B) Left: scatter plot of regional PLS1 scores (weighted sum of 15,633 gene-expression values) versus Z-statistics for case–control CBF differences in Dataset 1 (*r* = 0.415, *p* < 0.001). Right: scatter plot of regional PLS1 scores versus Z-statistics for case–control CBF differences in Dataset 2 (*r* = 0.313, *p* < 0.001).

**Validation dataset**

Subsequently, we validated this approach by associating the unsmoothed Dataset 1 (validation dataset) with gene expression data. Using partial least squares (PLS) regression, we found that the first PLS component (PLS1) explained 14.73% of the spatial variance in cerebral blood flow (CBF) based on the weighted gene expression pattern from the case–control difference map (after correction for spatial autocorrelation, *p* = 0.003; Fig. S5). The validation analysis identified a total of 7839 significant contributing genes (FDR‑corrected *p* < 0.05), of which the PLS1+ gene set contained 3192 genes and the PLS1− gene set contained 4647 genes. Compared with Dataset 1, 7459 overlapping genes were found (3062 from PLS1+ and 4397 from PLS1−); compared with Dataset 2, 2063 overlapping genes were found (732 from PLS1+ and 1331 from PLS1−). Overlap with the main gene set from this study (2187 genes) included 2032 genes, corresponding to an overlap rate of 92.91% (Fig. S6). Among these overlapping gene sets, the top-ranked genes consistently included *AK5*, *PTPRN2*, and *GRIN2B*. Collectively, these results demonstrate that the smoothing step had a limited impact on the findings and did not alter the key genes identified.

**Figure S5. PLS regression analysis and correlation analysis in validation dataset**


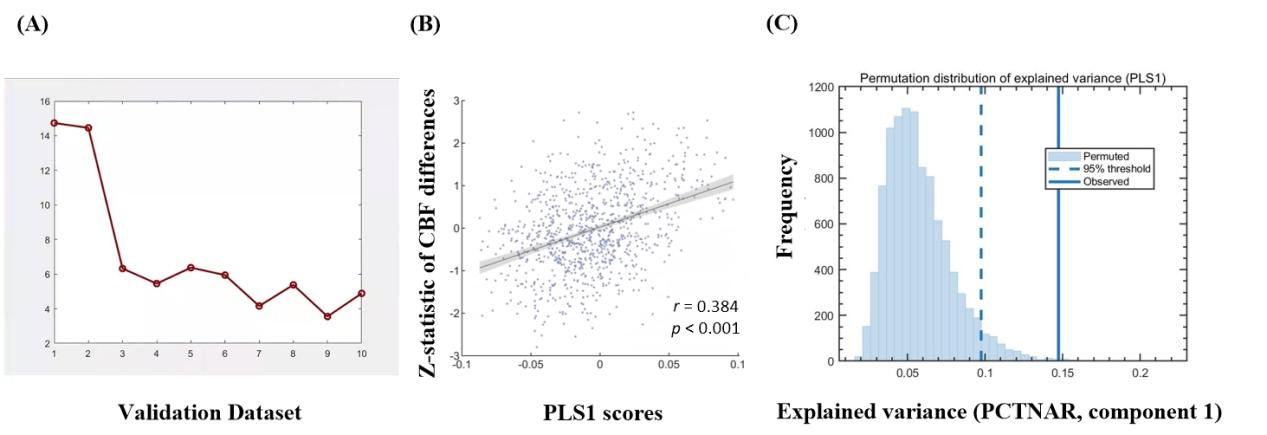


1. Explained ratios for the first 10 components from the PLS regression analysis. (B) Plot of regional PLS1 scores (weighted sum of 15633 gene-expression values) versus z-statistics for case–control CBF differences in validation dataset (*r* = 0.384, *p* < 0.001). (C) The permutation distribution of explained variance of the PLS1 model.

**Figure S6. Comparison of top-ranked and overlapping genes between validation and discovery datasets**

1. Comparison between validation dataset and dataset 1. (B) Comparison between validation dataset and dataset 2. (left panel: ranked PLS1 loadings; right panel: top-ranked and overlapped genes from two datasets). (C) Overlap of two shared gene sets: (dataset 1 vs dataset 2) and (validation dataset vs dataset 2)

**Table S1 Demographic and clinical characteristics in the meta-analysis studies**

| Study | subtype | Indicator | Scanner | Subjects(male/female) | | Mean age (SD) | |
| --- | --- | --- | --- | --- | --- | --- | --- |
|  |  |  |  | PD | HC | PD | HC |
| Barzgari et al. | / | CBF | 3T GE/8-channel head coil | 30(24/6) | 31(25/6) | 66.14(8.97) | 67.48(8.31) |
| Lin et al. | / | CBF | 1.5T GE/8-channel head coil | 20(6/14) | 22(7/15) | 63.30(6.40) | 59.90(6.00) |
| Wang et al. | PD-FOG | CBF | 3T GE/24-channel head coil | 30(15/15) | 30(16/14) | 64.13(6.72) | 62.90(7.56) |
| Wang et al. | PD-NFOG | CBF | 3T GE/24-channel head coil | 30(13/17) | 30(16/14) | 60.73(8.30) | 62.90(7.56) |
| Fernández-Seara et al. | / | CBF | 3T Siemens/12-channel head coil | 25(18/7) | 34(12/22) | 63.20(6.60) | 63.50(6.60) |
| Lin et al. | PD | CBF | 1.5T GE/8-channel head coil | 17(7/10) | 17(8/9) | 60.88(10.18) | 59.65(7.54) |
| Lin et al. | PDD | CBF | 1.5T GE/8-channel head coil | 17(6/11) | 17(8/9) | 63.71(8.54) | 59.65(7.54) |
| Wang et al. | / | CBF | 3T GE/8-channel head coil | 47(29/18) | 50(28/22) | 69.00(7.60) | 70.10(6.20) |
| Shang et al. | PD-L | CBF | 3T GE/8-channel head coil | 44(18/26) | 45(22/23) | 60.23(6.49) | 58.69(4.12) |
| Shang et al. | PD-R | CBF | 3T GE/8-channel head coil | 48(22/26) | 45(22/23) | 58.83(5.65) | 58.69(4.12) |
| Suo et al. | PD-NC | CBF | 3T Siemens/12-channel head coil | 17(10/7) | 36(15/21) | 54.00(8.20) | 53.70(7.30) |
| Suo et al. | PD-MCI | CBF | 3T Siemens/12-channel head coil | 22(9/13) | 36(15/21) | 53.80(8.50) | 53.70(7.30) |
| Suo et al. | ALL-PD | CBF | 3T Siemens/12-channel head coil | 39(19/20) | 36(15/21) | 53.89 | 53.70(7.30) |
| Laganà et al. | / | CBF | 1.5T Siemens/12-channel head coil | 26(22/4) | 18(11/7) | 65.60(8.25) | 66.85(8.00) |
| Al-Bachari et al. | PD-TD | CBF | 3T Philips/8/32-channel head coil | 21(15/6) | 34(16/18) | 67.90(7.20) | 67.40(7.60) |
| Al-Bachari et al. | PD-PIGD | CBF | 3T Philips/8/32-channel head coil | 24(19/5) | 34(16/18) | 70.00(7.60) | 67.40(7.60) |
| Al-Bachari et al. | ALL-PD | CBF | 3T Philips/8/32-channel head coil | 51(39/12) | 34(16/18) | 69.00(7.70) | 67.40(7.60) |
| Arslan et al. | PD-NC | CBF | 3T Philips/32-channel head coil | 26(16/10) | 15(11/4) | 60.15(8.98) | 58.67(6.30) |
| Arslan et al. | PD-MCI | CBF | 3T Philips/32-channel head coil | 27(21/6) | 15(11/4) | 64.00(8.14) | 58.67(6.30) |
| Chen et al. | PDD | CBF | 3T GE/8-channel head coil | 26(18/8) | 35(18/17) | 68.50(7.01) | 68.60(3.98) |
| Jia et al. | PD-NC | CBF | 3T Siemens/8-channel head coil | 27(15/12) | 25(11/14) | 63.11(9.27) | 59.44(5.77) |
| Jia et al. | PD-MCI | CBF | 3T Siemens/8-channel head coil | 27(16/11) | 25(11/14) | 62.59(6.61) | 59.44(5.77) |

Continued Table S1

| H-Y stage | UPDRS-III | duration(years) | Education(years) | | MMSE (SD) | | MoCA(SD) | |
| --- | --- | --- | --- | --- | --- | --- | --- | --- |
|  |  |  | PD | HC | PD | HC | PD | HC |
| 1.75(0.70) | 20.25(10.59) | 6.14(3.90) | 15.47(2.84) | 17.81(2.54) | / | / | / | / |
| 2.00(0.80) | 22.90(15.10) | 2.50(1.50) | 8.80(4.90) | 11.50(4.90) | 22.60(7.40) | 27.10(2.10) | / | / |
| 2.58(0.87) | 31.20(16.47) | 6.50(3.86) | / | / | 25.60(2.08) | 25.93(1.76) | / | / |
| 2.17(0.84) | 28.93(10.35) | 5.35(2.79) | / | / | 25.17(1.88) | 25.93(1.76) | / | / |
| 1.80(0.40) | 12.50(5.30) | 5.60(3.40) | / | / | 27.90(2.30) | / | / | / |
| 2 | 17 | 2.48(1.53) | 9.29(4.10) | 10.19(6.03) | 28.29(2.31) | 28.73(2.01) | / | / |
| 2 | 19 | 3.53(2.24) | 7.12(5.77) | 10.19(6.03) | 21.53(4.71) | 28.73(2.01) | / | / |
| 2 | 26.30(12.30) | 4 | 9 | 9 | 28 | 28 | / | / |
| 1.63(0.47) | 31.88(17.68) | 5.64(2.98) | 13.93(3.74) | 13.80(3.98) | 28.61(1.07) | 28.22(0.92) | / | / |
| 1.71(0.44) | 35.64(18.77) | 5.31(3.37) | 13.48(3.85) | 13.80(3.98) | 28.06(1.31) | 28.22(0.92) | / | / |
| 1.80(0.60) | 17.10(9.70) | 2.20(1.60) | 11.30(2.50) | 10.80(2.90) | 27.08(4.40) | 28.20(1.60) | 24.30(2.40) | 23.10(2.50) |
| 1.90(0.60) | 23.20(10.20) | 2.40(1.70) | 10.30(2.50) | 10.80(2.90) | 27.60(2.10) | 28.20(1.60) | 19.10(2.90) | 23.10(2.50) |
| 1.86 | 20.54 | 2.31 | 10.74 | 10.80(2.90) | 27.37 | 28.20(1.60) | 21.37 | 23.10(2.50) |
| 1.50(1.00) | 21.92(13.20) | 3(2) | / | / | / | / | 24.84(3.73) | 26.43(3.94) |
| 1.80(0.70) | 26.30(10.80) | 5.10(3.50) | / | / | / | / | 27.30(2.70) | 28.00(2.30) |
| 3.20(0.80) | 29.90(21.10) | 9.10(4.50) | / | / | / | / | 23.80(3.80) | 28.00(2.30) |
| 2.60(1.00) | 30.20(11.80) | 7.20(4.40) | / | / | / | / | 25.40(3.80) | 28.00(2.30) |
| 1.77(0.51) | 26.00(10.80) | 5.42(3.04) | 10.08(4.05) | 11.00(3.80) | 29.38(0.80) | 26.69(1.93) | 25.20(2.33) | 16.40(7.10) |
| 1.92(0.56) | 32.40(13.11) | 6.62(3.54) | 9.00(3.67) | 11.00(3.80) | 28.15(1.41) | 26.69(1.93) | 22.37(2.52) | 16.40(7.10) |
| 2.50 | 34.72(6.50) | 6.12(4.57) | 6 | 9 | 14.12(4.11) | 28.75(1.49) | 14.15(3.48) | 27.28(1.17) |
| 1.88(0.50) | 23.82(7.38) | 3.71(2.96) | 13.37(2.91) | 12.08(2.91) | / | / | / | / |
| 1.71(0.68) | 21.38(9.53) | 3.65(2.86) | 7.78(4.27) | 12.08(2.91) | / | / | / | / |

PD, Parkinson’s Disease; HC, Healthy control; CBF, cerebral blood flow; MMSE, Mini-Mental State Examination; MoCA, Montreal Cognitive Assessment; UPDRS, Unified Parkinson’s Disease Rating Scale; H-Y stage, Hoehn-Yahr staging; PD-NC, PD-Normal Control; PD-MCI, PD-Mild Cognitive Impairment; PDD, PD-Dementia; PD-FOG, PD-Freezing Of Gait; PD-TD, PD-Tremor Dominate; PD-PIGD, PD-Postural Instability and Gait Disorder; PD-L, PD-Left sided symptom dominance; PD-R, PD-Right sided symptom dominance.
